# Uniportal VATS Approach in Esophageal Cancer – How to Do It Update

**DOI:** 10.3389/fsurg.2022.844796

**Published:** 2022-03-25

**Authors:** Hasan Batirel

**Affiliations:** Thoracic Surgery Unit, Memorial Sisli Hospital, Istanbul, Turkey

**Keywords:** uniportal and tubeless video-assisted thoracic surgery, esophagectomy, side to side anastomosis, videothoracoscopic surgery, Ivor Lewis minimally invasive esophagectomy

## Abstract

The adoption of minimally invasive esophagectomy has been used for over a decade, and the chest part is evolving into a uniportal video-assisted thoracoscopic surgery (VATS) approach. Uniportal esophageal mobilization and anastomosis have many peculiar aspects, which include placement of the incision, alignment of instruments, and anastomosis. The incision is placed over the sixth intercostal space posterior axillary line. The esophagus is usually encircled at the level of the inferior pulmonary vein. The use of curved suction helps in the retraction of the esophagus and the exposure of the left main bronchus deep in the mediastinum. For intrathoracic anastomosis in Ivor Lewis esophagectomy, a completely side-to-side linear-stapled anastomosis is preferred. This anastomotic technique results in a long stapler line. The correct alignment of tissues and adequate anastomotic circumference are of utmost importance to prevent leaks or strictures. Perioperative and oncologic results in several series with uniportal VATS, esophageal mobilization, and anastomosis are comparable with open or other types of minimally invasive esophagectomy. Uniportal VATS for esophagectomy is feasible and fast with good results.

## Introduction

The adoption of minimally invasive techniques in esophageal cancer resection has been slower than in other surgeries. This was mainly due to the lack of any benefit from minimally invasive approaches in terms of major morbidity and mortality in the initial reports (1). Additionally, many authors showed that the learning period of minimally invasive esophagectomy (MIE) is much longer than thought. White et al. reported no mortality and leak in 40 patients after 130 minimally invasive Ivor Lewis esophagectomies (2). In the first 130 patients, mortality was four and the leak rate was 8%.

A randomized multicentric study by Biere showed significantly less pulmonary morbidity following MIE compared with an open approach (3). Fewer cardiopulmonary complications were also reported in a randomized control trial that compared robotic and open esophagectomies (4). We reported our uniportal video-assisted thoracoscopic surgery (VATS) technique in esophageal cancer in 2017 and presented our results in the first 18 patients in the 2019 European Society of Thoracic Surgeons meeting (5, 6).

The classical VATS approach for esophageal cancer utilizes three to four incisions (7). Robotic esophagectomy utilizes four ports in the chest and low-pressure carbon dioxide insufflation (8). There are challenges in the technical manipulations of uniportal VATS because esophageal mobilization or anastomosis is more difficult as anatomic structures in the deep posterior mediastinum are located in narrow spaces. We have previously demonstrated that uniportal VATS esophageal mobilization or esophageal resection/anastomosis is feasible (5). In this article, we describe technical modifications to our initial uniportal technique for esophageal cancer.

## Technical Modifications in Uniportal MIE

Our uniportal technique for esophageal cancer resection has been described in detail in two previous articles (5, 9). We have made changes to our technique and these novel additions are described in the following sections.

### Positioning and Placement of the Incision

In the beginning, we were performing the procedure in a left lateral decubitus position with a 30° tilt to the ventral side. The left lateral decubitus positioning did not change; however, in cases with a narrow chest and emphysematous lungs, we increased this tilt to 45° to facilitate better exposure of the posterior mediastinum.

**Figure 1:**
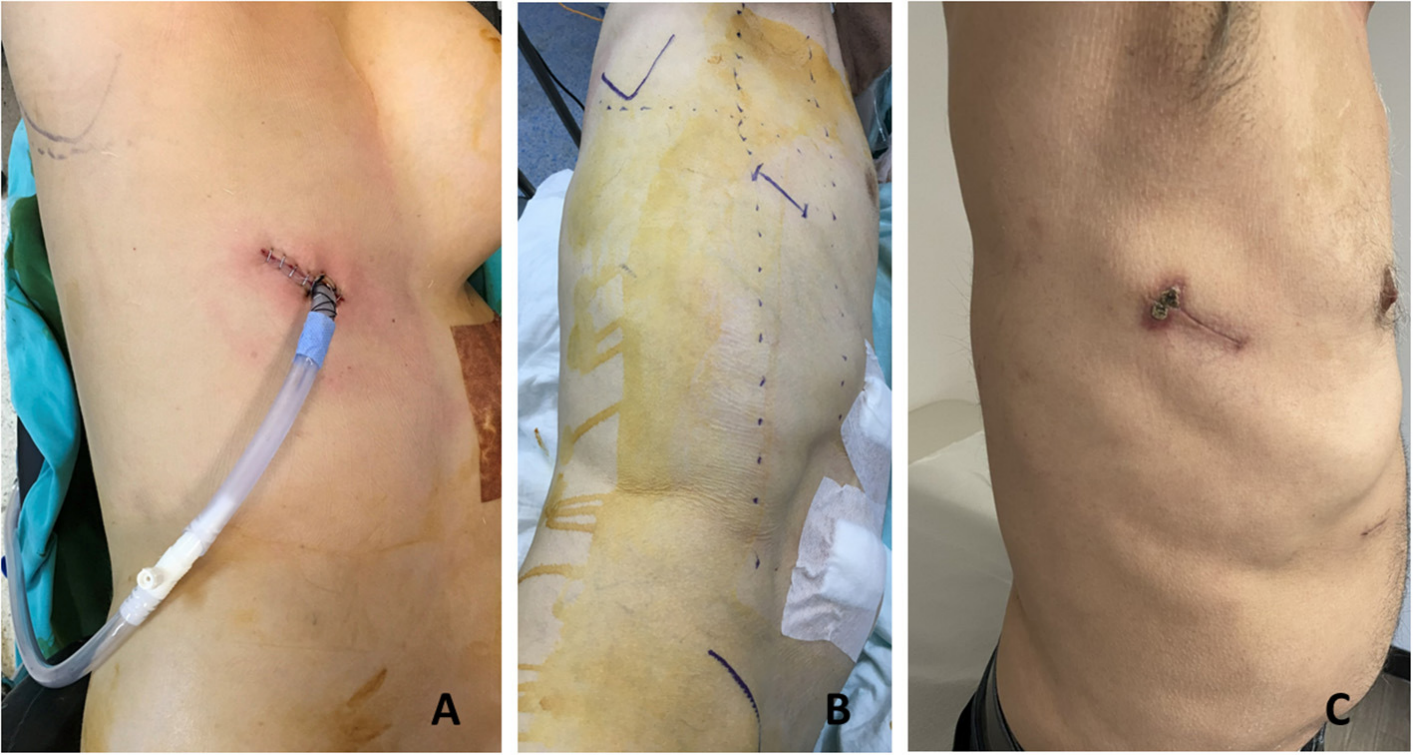
The uniportal incision is made on the sixth intercostal space posterior axillary line. **(A)** In a 55-year-old female patient, we placed our incision between the anterior and posterior axillary lines. **(B)** Preoperative planning in a 57-year-old man. **(C)** One month appearance of a uniportal incision for esophageal mobilization in a 69-year-old patient.

In our initial approach, we placed our incision on the fifth or sixth intercostal space between the posterior and anterior axillary lines. But we noticed that, in most of the patients, the sixth intercostal space incision behind the posterior axillary line was the best access point with comfortable exposure of the distal esophagus and hiatus (Figure 1). From this access point, it is possible to see the whole posterior mediastinum from below. The sixth intercostal space incision also allows more flexible movement of instruments at the higher parts of the chest. The anterior end of the incision lies on the posterior axillary line and the chest tube is placed on the anterior side of the incision. The chest tube is better aligned to the posterior mediastinum alongside the conduit and rests on the diaphragm providing better drainage. A 4 cm incision appears to be enough for all the procedures and if the tumor is not very hard and bulky, removal of the specimen in a bag through this incision is easy and not traumatic.

### Intraoperative Manipulations

**Figure 2:**
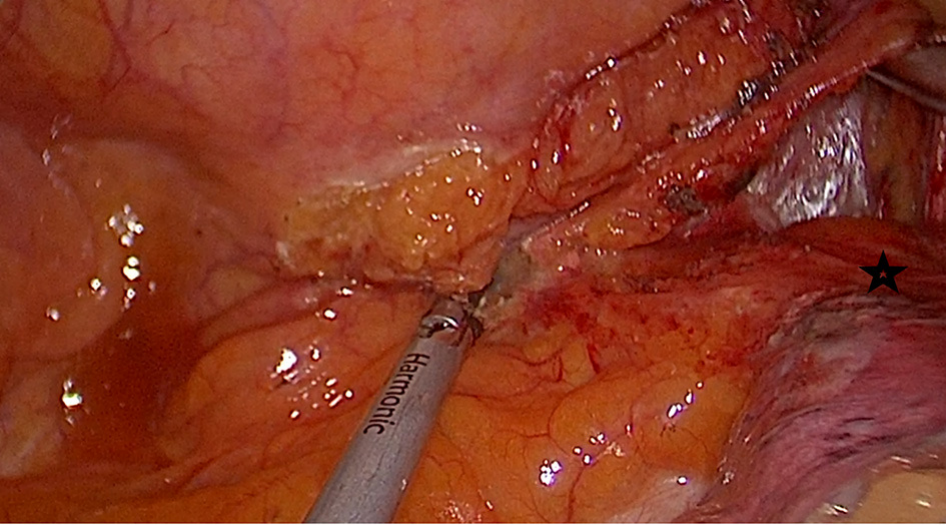
Mobilization of the esophagus starts at the lower end. Pericardium (black star) posterior to the inferior pulmonary vein is the anterior plane for esophageal mobilization.

Initial dissection is started at the inferior pulmonary ligament, and once lymph nodes are lifted to the esophagus, the pericardium posterior to the inferior pulmonary vein is a good guide to develop an avascular plane anterior to the esophagus (Figure 2). If the tumor is located at this level, it may be difficult to go around the esophagus due to tumor size, adhesions, and fibrosis secondary to neoadjuvant treatment. In such a situation, another level of dissection is the subcarinal lymph node plane. Once the subcarinal lymph node is mobilized from the right main bronchus, the pericardial plane anterior to the lymph node is relatively avascular. The left side of the lymph node can be lifted from the left main bronchus, and the esophagus, which lies closer to the right side of the chest at this level, can be encircled easily. If the subcarinal lymph node is large, fibrotic, and would not allow a comfortable dissection, it may be removed later. In such a circumstance, the posterior plane to the subcarinal lymph node can also be used for esophageal mobilization. The careful division of small vessels between the subcarinal lymph node and esophagus is important to prevent troublesome bleeding. In a rare case of large and long middle-distal esophageal tumors post chemoradiation, both of these planes may be too fibrotic or the tumor may be too large preventing a safe exposure. In those situations, mobilization of the esophagus at the azygos vein or the hiatal level can be preferred; however, in most of those cases, an open approach would be a safer alternative to avoid excessive bleeding or bronchial injury.

Once the esophagus is encircled with a 2-cm-thick Penrose drain, it can be easily retracted anteriorly and posteriorly. Mobilization of the esophagus is straightforward after this maneuver. The azygos vein is divided. The esophagus is mobilized *en bloc* with the periesophageal lymph nodes. The aortic branches should be divided carefully without traction. It is important to keep close to the esophagus during mobilization from the membranous side of the trachea. The level of upper mediastinal dissection of the esophagus is decided based on the tumor location, mediastinal radiation, and intrathoracic or cervical anastomosis.

### Reconstruction/Anastomosis

In the case of a minimally invasive Ivor Lewis esophagectomy, intrathoracic anastomosis is a very important stage of the operation. Our anastomotic technique has evolved, and we now put much more emphasis on the tissue quality of the esophagus, the circumference of the anastomosis, and the approximation of the stomach and the esophageal walls.

We avoid an intrathoracic anastomosis if the esophageal wall does not look healthy secondary to significant dilation or neoadjuvant radiation. In some patients, tumor obstruction causes chronic dilation of the proximal esophagus and usually, a healthy esophageal wall can only be found at the cervical level. This can be decided by reviewing tumor location, dilation on preoperative CT scans, and radiation fields. Five centimeters of healthy esophagus is needed to perform a side-to-side completely stapled anastomosis.

**Figure 3:**
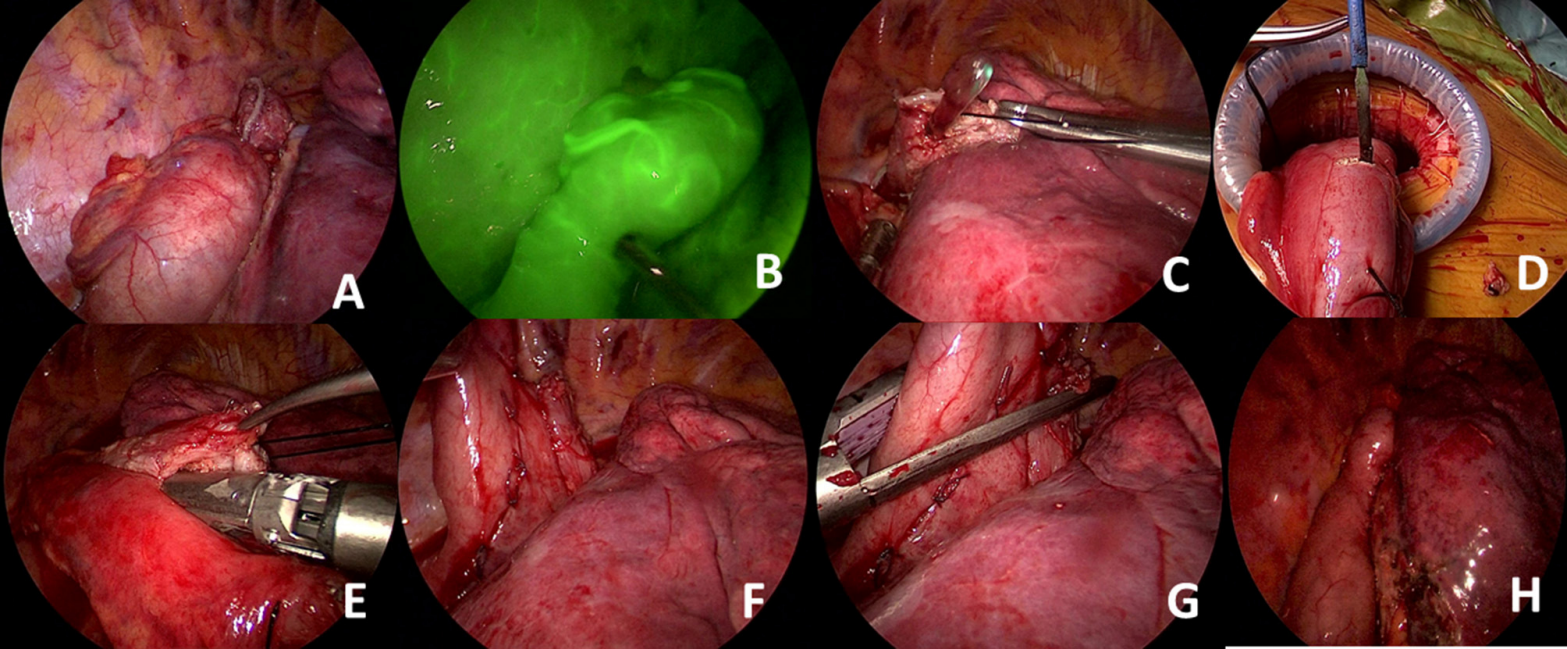
**(A)** The ends of the esophagus and stomach conduit are prepared and aligned correctly. **(B)** Gastric perfusion can be assessed with indocyanine green. Excellent perfusion of the gastric conduit is seen. **(C)** An esophagotomy is performed in the esophageal tip and the nasogastric tube is visualized. **(D)** A small gastrostomy is performed for placement of the thicker stapler leg. **(E)** Anastomosis at the posterior wall is performed by combining esophageal and gastric tips using a 60-mm-thick tissue stapler. **(F)** The esophageal and gastric walls can be clearly visualized. (**G)** Side placement of the thick tissue stapler to complete the anastomosis. **(H)** Visualization of final alignment of the conduit and anastomotic line.

We provide here a brief description of side-to-side completely stapled intrathoracic anastomosis (Figure 3). A no.1 silk suture is placed at the stapler line of the esophagus. The esophageal stump is cut under the staple line and mucosa is visualized. A nasogastric tube is pushed out of the opening. Then the stomach conduit is pulled out of the port incision. A gastrotomy is made 3–4 cm distal to the tip of the stomach conduit. Another no. 1 silk suture is placed at the tip of the stomach for retraction. The thicker leg of a 60-mm-thick tissue endoscopic stapler is placed inside the stomach. The stapler is advanced inside the chest while applying gentle traction to both silk sutures. The thin leg of the stapler is advanced inside the esophagus taking the nasogastric tube as a guide. Once both legs are inside the esophagus and stomach, the nasogastric tube is completely removed. The edges of the esophagus and stomach are aligned equally and the stapler is fired to form the posterior side of the anastomosis. Then, two firings of the 60 mm endoscopic stapler complete the side esophagogastrostomy.

This anastomotic technique is easy to perform and usually lasts 10–15 min. However, there are important details. In the classical VATS approach, a circular stapled anastomosis is performed using a 28 mm stapler. Based on the circumference formula of a circle (C = 2πr; C = circumference, π = 3.14, *r* = radius), the circumference of such an anastomosis is approximately 8.8 cm (8.792 = 3 × 3.14 × 1.4). A 60 mm stapler forming the posterior side of the anastomosis gives us a circumference of 12 cm. However, we do not close the immediate edge, and the endoscopic linear stapler legs are 10–15 mm wide. Thus, it is important to measure correctly and leave at least 8 cm of circumference to prevent postoperative strictures. In our series, we have noticed a stricture rate of 30% (unpublished data) with this anastomotic technique which is much higher than circular anastomosis. Most of those strictures were secondary to the too low firing of the side staplers.

**Figure 4:**
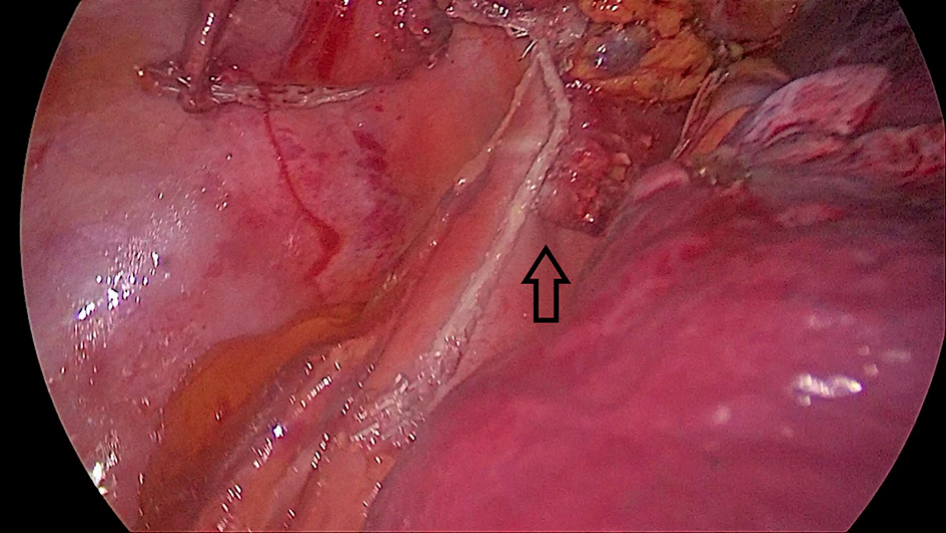
Misalignment of the stomach and esophageal walls (black arrow). This patient had a late leak and left main bronchial fistula that healed in 6 months after double stenting of the trachea and the esophagus.

Another important issue is the correct alignment of stapler lines when side staplers are fired. In some cases, anterior and posterior stapler lines are misaligned (Figure 4). This may lead to poor healing or fistulas to the pleura or even to the lung tissue.

**Figure 5:**
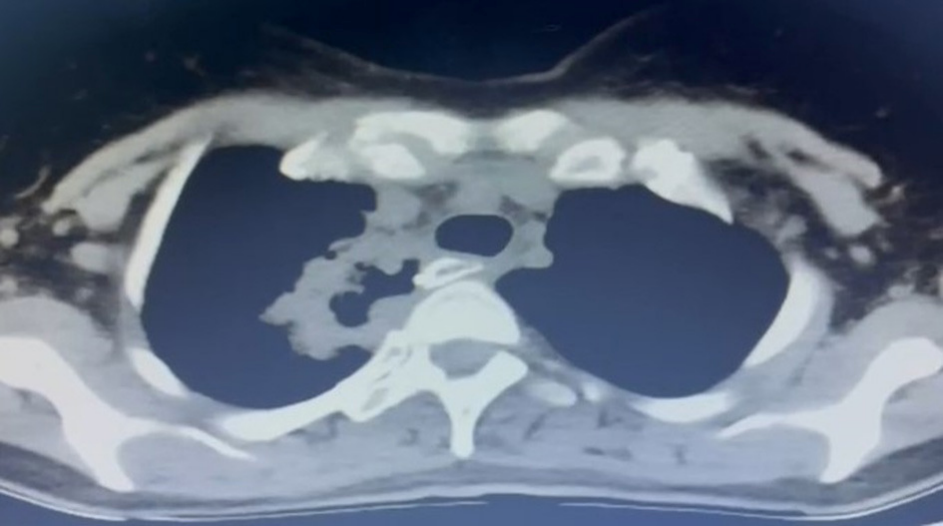
A late fistula (3 weeks after esophagectomy) to lung parenchyma from the stapler line in a 42-year-old female patient. The patient had preoperative chemoradiation and there was no pleural contamination.

The third issue is that a long stapler line also increases the risk of small healing defects. We have experienced lung abscesses secondary to stapler line fistulas in two patients without any pleural contamination (Figure 5). Both patients were chemoradiated and lung abscesses became clinically evident 3 weeks after esophagectomy. Covering the stapler line with omentum or pleura may prevent such a complication in high-risk patients.

## Discussion

The uniportal VATS approach for esophagus cancer is still in its early stages. It is important to demonstrate that the technique is reproducible with comparable perioperative and oncological results to open or other minimally invasive approaches. In this article, the modifications to the initial uniportal VATS esophageal cancer technique have been described in detail.

This comparison can be analyzed in four topics, namely duration of surgery, complete resection rates, and lymph node yield and anastomotic leak. We know that Biere et al. compared 59 prone-position MIE with 56 open esophagectomy patients in their prospective randomized trial (3). There was significantly less pneumonia (12 vs. 34%, respectively, *p* = 0.005), shorter hospital stays (11 vs. 14 days, respectively, *p* = 0.044), and less bleeding (200 vs. 475 ml, respectively, *p* < 0.001). Complete resection rate, total lymph node yield, mortality, and anastomotic leak rates were similar (3). In the same study, pain measured with a visual analog score was much less in the MIE group (*p* = 0.001). Robotic esophagectomy resulted in much less median blood loss (400 vs. 568 ml, *p* < 0.001), a lower percentage of pulmonary complications (RR.54; 95% CI, 0.34–0.85; *P* = 0.005) and cardiac complications (RR.47; 95% CI, 0.27–0.83; *p* = 0.006), and lower mean postoperative pain (visual analog scale, 1.86 vs. 2.62; *p* < 0.001) compared to open esophagectomy in a randomized trial of 112 patients (4). Additionally, functional recovery at postoperative day 14 was better in the robotic esophagectomy group along with better quality of life score at discharge and 6 weeks post-discharge. In the 3-year follow-up data of a randomized trial by Straatman et al., while overall survival, disease-free survival, and recurrence were not different between MIE and open esophagectomy groups, there was much less pulmonary infection in the MIE group (9 vs. 29%, respectively, *p* = 0.005) (10).

A recent meta-analysis comparing MIE (*n* = 4,948) with open esophagectomy (*n* = 8,321) revealed fewer pulmonary complications (OR = 0.56; CI = 0.41–0.78; *p* < 0.001), shorter hospital stays (SMD = −0.51; CI = −0.78 to −0.24; *p* < 0.001), and less bleeding (SMD: −1.44; CI = −1.95 to −0.93; *p* < 0.001) with MIE, confirming the outcomes of previous studies (11).

It can be presumed that fewer incisions would lead to less trauma; thus, a uniportal VATS approach should have less likelihood of causing intercostal neuralgia. A small study comparing biportal VATS with four-port VATS showed no difference in perioperative and oncologic outcomes between the two techniques (12). A similar study by Lee et al. comparing multiportal with uniportal VATS in esophageal cancer found a similar duration of surgery, amount of bleeding, hospital stay, and total lymph node count. The only difference was significantly less pain on postoperative day 7 in the uniportal VATS group (13).

Reports on uniportal VATS in esophageal cancer are limited. A 12-patient series by Nachira et al. (14) reported an average duration of 105 ± 21 min in the chest part and 10.4 ± 3.9 lymph nodes. Wang et al. (15) reported 44 esophageal mobilizations with a uniportal VATS technique. The average duration was 163 ± 16 min and the lymph node yield was 24. In both of these series, anastomosis was performed in the neck (14, 15). In our experience, if the proximal margin of cancer was located distal to the carina, our intention was to perform a high intrathoracic anastomosis. If the proximal end of the tumor was higher than the carina then a left neck anastomosis was preferred. In our initial experience on 18 patients who underwent uniportal VATS esophagectomy (16 cases with Ivor Lewis) for esophageal cancer showed that there was no perioperative mortality (6). The average number of lymph nodes was 23 ± 8, and the mean duration of VATS was 82 ± 22 min. Eight patients had preoperative chemoradiation. Three patients had leaks, two occurred in the chest (1 late; 22 days after surgery) and one in the neck. Our current experience in 40 patients is similar with a median thoracic VATS duration of 90–100 min including anastomosis (10–15 min) and a lymph node yield of 20–25.

One of the dreaded complications is an anastomotic leak in the chest. Despite all the experience and various techniques, there are technical reasons which can be avoided, whereas patient-related factors cannot be influenced in all cases. For a successful anastomosis, Sweet's principles of 1946 are still valid. Even in the minimally invasive era, principles to be followed are no tension in the anastomosis, atraumatic handling of anastomosis areas, well-placed separate sutures, and circumferential mucosal approximation (16). A circular stapler or the OrVil technique are the most commonly used techniques in MIE (7). We prefer a side-to-side completely stapled anastomosis as it is easier and faster to perform, and we cause almost no trauma to the anastomosis parts. There are two important details with this technique, the location and width of the anastomosis. For tension-free anastomosis, esophagus and stomach cuts should be made after measurement. The stomach conduit should be interposed in the chest to identify the site it can comfortably reach before any division is made on the esophagus. If the side closure of the anastomosis is too low, a stricture is inevitable in the side-to-side anastomosis, and before firing the stapler, a correct estimation of the anastomotic circumference should be made. In our experience with uniportal VATS Ivor Lewis esophagectomies, the leaks (three in total) were due to various reasons. In one of the patients, prolonged hypotension was an important contributor; in another patient, the anastomosis was in the radiation field and the esophagus was not healthy in a retrospective look. Thus, whatever the anastomotic technique is used, the Sweet principles and optimization of patient-related factors are crucial for good results.

In a recent meta-analysis by Achaempong et al., hybrid, totally MIE, and robotic esophagectomy were compared and there was no difference in overall survival between the techniques at 3 years (17). Patel et al. also showed no difference in the 5-year overall and disease-free survival between MIE and open esophagectomy (18).

In conclusion, uniportal VATS mobilization or esophagectomy is feasible with comparable perioperative and oncologic outcomes. The results are similar to other MIE or open approaches and can present the patients with an alternative of a single small incision for the same surgical procedure.

## Data Availability Statement

The original contributions presented in the study are included in the article/supplementary material, further inquiries can be directed to the corresponding author/s.

## Ethics Statement

The studies involving human participants were reviewed and approved by Marmara University Hospital, Istanbul, Turkey. The patients/participants provided their written informed consent to participate in this study.

## Disclosure

HB is a consultant for Johnson and Johnson and receives fees and honoraria.

## Author Contributions

The author confirms being the sole contributor of this work and has approved it for publication.

## Conflict of Interest

The author declares that the research was conducted in the absence of any commercial or financial relationships that could be construed as a potential conflict of interest.

## Publisher's Note

All claims expressed in this article are solely those of the authors and do not necessarily represent those of their affiliated organizations, or those of the publisher, the editors and the reviewers. Any product that may be evaluated in this article, or claim that may be made by its manufacturer, is not guaranteed or endorsed by the publisher.
